# Dynamic neurocognitive adaptation: childhood and adult-midlife engagement associated with later-life brain structure and cognition in older adults with and without mild cognitive impairment

**DOI:** 10.1007/s11682-026-01122-0

**Published:** 2026-03-21

**Authors:** Filippo Cieri, Jessica Z. K. Caldwell, Dietmar Cordes, Chad L. Cross

**Affiliations:** 1 Department of Neurology, Cleveland Clinic Lou Ruvo Center for Brain Health, Las Vegas, NV USA; 2 University of Madison Winsconsin, Madison, WI USA; 3 University Bolder Colorado, Bolder, CO USA; 4 School of Veterinary Medicine, Texas Tech University, Amarillo, TX USA

**Keywords:** Dynamic Neurocognitive Adaptation (dNA), resilience, resistance, aging, cortical thickness, brain volume, cognition, mild cognitive impairment, Alzheimer’s disease.

## Abstract

**Background:**

Resilience in aging—the capacity to maintain cognition and function despite neuropathology—has been described through cognitive reserve, brain reserve, and maintenance. The *dynamic Neurocognitive Adaptation* (dNA) framework expands these constructs by defining resilience as a lifelong process of adaptive engagement across cognitive, physical, creative, and social domains that shape neural integrity and cognitive outcomes over time.

**Methods:**

Fifty-eight older adults (39 cognitively normal, 19 with mild cognitive impairment) completed neuropsychological testing, amyloid assessment, and structural MRI. The dNA scale quantified engagement across seven life-course time windows. Hierarchical multiple regressions examined domain- and time-specific associations between dNA scores and cortical thickness or regional volumes (FreeSurfer 7.3.2), controlling for age, sex, education, and diagnosis. Exploratory mediation and moderation models tested indirect and interaction effects of demographic and diagnostic factors.

**Results:**

Childhood (Time Window 1) emerged as a sensitive period: higher cognitive engagement is associated with stronger semantic fluency, and physical engagement is linked to a better episodic learning. Creative and physical engagement during childhood related to larger anterior and posterior cingulate and temporal-pole volumes. In adulthood and midlife (Time Windows 3–5), greater engagement was associated with thicker right lateral orbitofrontal cortex and larger lingual volumes. No mediation or moderation by sex, education, or diagnosis was observed.

**Conclusions:**

Childhood and midlife emerge as sensitive periods linking multidomain engagement with later-life brain structure and cognition. The dNA framework provides a multidimensional, time-resolved model of resilience, illustrating how lifelong adaptive behaviors support neural integrity and cognitive health across aging and Alzheimer’s-disease risk.

**Supplementary Information:**

The online version contains supplementary material available at 10.1007/s11682-026-01122-0.

## Introduction

The Collaboratory on Research Definitions for Reserve and Resilience in Cognitive Aging and Dementia (Stern et al; 2020, 2023) defined resilience as “any concept that relates to the capacity of the brain to maintain cognition and function with aging and disease”. Such concepts include cognitive reserve, brain reserve, and brain maintenance. Contemporary models, such as the integrative framework proposed by the Reserve & Resilience Operationalization in Aging and Neurodegenerative Disease (ReROAND) working group, integrate these distinctions to focus on the overarching dynamic of neurocognitive resilience (Stern et al., 2020; 2023) Building upon this, we propose the concept of dynamic Neurocognitive Adaptation (dNA) to further explore this dynamic perspective.

In physical and material sciences resilience is defined as “the ability of a material to absorb energy when deformed and recover when the load is removed” (Oxford English Dictionary; Merriam-Webster). In biological systems, a closely related concept is adaptation: the process first described by Darwin (1859-2009) as organisms becoming fitted to their conditions of life and later refined by Mayr (1982) as a dynamic, never perfect, but always relative adjustment to changing environments. This principle underlies our concept of dynamic Neurocognitive Adaptation (dNA), extending evolutionary adaptation to the neurocognitive domain across the life course. Importantly, adaptation is particularly relevant to the brain, which is characterized by remarkable plasticity (Hebb, 1949; Kolb & Whishaw, 1998; Pascual-Leone et al., 2005), defined as the capacity to modify its structure and function in response to experience, environment, and injury, providing a biological substrate for lifelong adjustment and adaptation.

From this perspective, resilience, resistance, and maintenance (Aron, 2022; Lissek, 2023; Liu et al., 2022; Park & Reuter-Lorenz, 2009) represent distinct yet related approaches to understanding adaptive mechanisms. As mentioned, In materials science, resilience denotes a return to the original state once an external load is removed, recovery from strain or deformation. Living organisms have different response(s). Especially in humans, any perturbation—illness, injury, stress, or enrichment—alters the system’s subsequent state and history; the organism does not “reset” to a pristine baseline (original state). Adaptation is a continuous, history-dependent process of adjustment to internal and external demands that unfolds across the lifespan, with or without disease. Whereas resilience in materials typically emphasizes recovery after stress, with the goal of returning to the original state, biological adaptation is a dynamic, life-centered process that unfolds over time, continuously adjusting to internal and external demands with or without disease or insult. This dynamic quality is essential in neurocognitive aging, where change is a fundamental and an intrinsic property of biological systems, not shared in the physics of materials, where change is typically an external perturbation (strain, deformation).

Adaptation could provide a complementary, biologically grounded and time-oriented concept, capturing the continuous, dynamic adjustments that characterize human development, aging, and disease. The dNA framework conceptualizes adaptation as a time-oriented, multidimensional process that operates across the life course, in human and non-human organisms. dNA situates adaptation as the foundational mechanism through which resistance and resilience manifest in neurocognitive aging, thereby extending and complementing existing reserve–resilience models.

The dNA builds on and extends existing models, with foundations in philosophical traditions that enrich neuroscientific approaches to brain health across the lifespan. Dual-aspect monism, first formulated by Spinoza (Spinoza, 1677/1985), Princeton University Press.holds that mind and body are two attributes of a single substance. This view was further developed by Russell (1921) (1921/2001 as per references in bibliography), Pauli and Jung (1955) (1955/2014 as per reference in bibliography), and more recently by Atmanspacher (2012), and Solms (2021), converging on the idea that subjective experience and neural mechanisms are complementary aspects of a single underlying reality. Building on this perspective, the dNA highlights the temporal dimension and multidimensional engagement—cognitive, physical, creative, and social—that scaffold resistance and resilience across the life span. A central aim of applying this framework is to better clarify what we mean when we refer to environmental protective factors for resilience, and when in the life course such factors may be most influential.

The dNA scale is a brief, self-report questionnaire designed to assess lifetime engagement in cognitively, physically, creatively, and socially enriching activities across multiple life-course time windows. The scale has been previously developed and validated in a large independent sample (Cieri et al. 2025a, b) and provides a concise measure of domain-specific engagement across childhood through aging.

In this study, we applied the dNA framework to examine whether experiences across different life stages—both early and later—relate to *neuro* (imaging) measures of brain volume and cortical thickness (CT), and *cognitive* outcomes, including episodic memory and semantic fluency, in cognitively normal (CN) and mild cognitive impairment (MCI) participants. To characterize how dNA relates to neural and cognitive aging, we ran complementary mediation and moderation analyses. Mediation evaluated whether regional CT or volume statistically accounted for associations between dNA engagement and cognition, adjusting for age, sex, education, diagnosis (and Total Intracranial Volume, TIV, for volume models). Moderation assessed whether sex, education, or diagnosis modified dNA–brain, brain–cognition, or dNA–cognition links using hierarchical regressions with interaction terms and post-hoc probing of significant effects.

Based on the dNA framework and prior literature on reserve and resilience, linking lifelong engagement to cognitive aging and brain integrity, we first hypothesized that cognitive and physical engagement would show the strongest associations with later-life cognitive performance and structural brain measures. Second, specifically related to the effect of time, we hypothesized that midlife engagement would represent a particularly sensitive period, such that dNA scores during mid-adulthood time windows would exhibit the most robust associations with later-life cognition and brain structure. Finally, while our primary hypotheses focused on cognitive and physical domains and on midlife engagement, associations across the remaining domains and developmental periods were examined in an exploratory manner to identify potential contributions to variability in neuropsychological and structural MRI outcomes.

## Methods

### Participants

This study was authorized by the Cleveland Clinic Institutional Review Board (Study #15–888) and conducted in accordance with the principles outlined in the Declaration of Helsinki. The sample included 58 older adults (39 CN individuals and 19 MCI individuals) from the Center for Neurodegeneration and Translational Neuroscience (CNTN), a National Institutes of Health (NIH)-funded Center of Biomedical Research Excellence (COBRE) initiative. Although the COBRE - CNTN cohort includes approximately 350 participants, at the time of the current study, the dNA scale had been administered to a subset of participants only. The analytic sample therefore comprised all older adults with complete dNA, neuropsychological, amyloid assessment, and structural MRI data, yielding a final sample of 58 participants. This approach ensured maximal use of available data relevant to the study hypotheses and avoided selective inclusion based on outcome measures. All participants provided written informed consent and completed a comprehensive demographic, diagnostic, and neuropsychological evaluation, including the Montreal Cognitive Assessment (MoCA), Mini-Mental State Examination (MMSE), episodic memory testing with the Rey Auditory Verbal Learning Test (RAVLT), semantic fluency (vegetables and animals), the dynamic Neurocognitive Adaptation (dNA) scale, amyloid status assessment, and structural MRI for cortical thickness (CT) and volumetric analyses.

Participants were classified as CN, or MCI based on the standardized diagnostic criteria of the Center for Neurodegeneration and Translational Neuroscience (CNTN) and COBRE initiatives, requiring consensus diagnosis by at least two clinicians (neurologist/neuropsychologist). CN participants were required to have: (1) no cognitive complaints (self/informant); (2) a Clinical Dementia Rating (CDR) global score of 0; (3) a normal neurological exam; (4) performance within 1.0-1.5 standard deviations of demographically adjusted norms on a comprehensive neuropsychological battery; and (5) no functional impairment in instrumental activities of daily living. MCI participants met the core clinical criteria for MCI due to AD: (1) subjective cognitive concern (confirmed by an informant); (2) objective impairment in one or more cognitive domains (≥ 1.5 SD below norms on neuropsychological testing); (3) essentially preserved functional abilities (CDR = 0.5); and (4) absence of dementia. All participants underwent amyloid PET imaging for research stratification. Exclusion criteria for all groups included history of other neurological, major psychiatric, or systemic illnesses that could affect cognition.

### Dynamic neurocognitive adaptation (dNA) scale

All participants completed the dNA scale, measuring lifetime engagement in four lifestyle domains: Cognitive (COG), Physical (PHY), Creative (CRE), Social (SOC). Each domain was assessed across seven life periods, or time windows (TWs): TW1 = 0–10 years (childhood); TW2 = 11–20 years (adolescence); TW3 = 21–30 years (youth); TW4 = 31–40 years (adulthood); TW5 = 41–50 years (middle age); TW6 = 51–64 years (senior); TW7 = + 65 years (old age). TW7 (≥ 65 years) was not included in the present analyses because it is contemporaneous with, or proximal to, the period in which later-life outcomes were assessed, increasing interpretive ambiguity (e.g., reverse causation) in this retrospective design. Therefore, analyses were restricted to TW1-TW6 to emphasize engagement occurring prior to the outcome assessment period. Each domain includes multiple items per TW (for details see Cieri et al. 2025a, b). Domain-specific mean scores were computed across items for each TW (e.g., CRE3 = mean of creative domain in TW3). All TW-domain scores were standardized as Z-scores using SPSS ZQ transformation to allow comparability across individuals. This standardization ensured that predictors were on a common metric, enabling direct comparison of standardized coefficients, minimizing bias from domains with different numbers of items, reducing scale-related distortions in comparisons.

### Neuropsychological assessment

All participants completed a comprehensive neuropsychological battery. For the present study, we focused on episodic memory and semantic fluency as primary cognitive outcomes, given their established sensitivity to cognitive aging and AD pathology (Henry et al., 2004: Petersen, 2004; Salmon & Bondi, 2009). Episodic memory was assessed using the Rey Auditory Verbal Test (RAVLT, Rey, 1964). Semantic fluency was evaluated via category fluency tasks (Animals and Vegetables; Benton, 1968). These measures were selected based on their theoretical relevance to reserve and resilience frameworks and because they were available in a complete, harmonized form for the entire analytic sample.

### Amyloid status comparison

An independent group comparison of amyloid positivity (binary classification: 0 = negative, 1 = positive) was conducted between CN and MCI participants. A Chi-Square Test of Independence was performed to assess whether the distribution of amyloid-positive cases significantly differed by diagnostic group.

### Structural MRI acquisition and analysis

MRI data were acquired on a 3-Tesla Siemens Skyra (Siemens Medical Solutions, Erlangen, Germany) using a 32-channel head coil. High-resolution T1-weighted structural images were collected with an MPRAGE sequence (TR = 2300 ms, TE = 2.98 ms, flip angle = 9°, voxel size = 1 mm isotropic, field of view = 256 mm, 176 sagittal slices). All scans underwent routine on-scanner QA and visual inspection for motion or artifacts prior to processing. T1-weighted images were processed with FreeSurfer v7.3.2 (https://surfer.nmr.mgh.harvard.edu/) using the standard recon-all pipeline (intensity nonuniformity correction, skull stripping, Talairach alignment, white/gray matter segmentation, and cortical surface reconstruction; see Fischl, 2012). Cortical parcellations followed the Desikan–Killiany atlas, yielding 34 cortical regions per hemisphere (Desikan et al., 2006). For each participant, regional cortical thickness (in millimeters) was computed as the distance between the pial and white matter surfaces and averaged within each atlas parcel. Thickness values were exported at the parcel level for statistical analysis. Because cortical thickness has been shown to better characterize neurodegeneration in AD than cortical volume (Schwarz et al., 2016), it served as our primary morphometric outcome. In parallel, regional gray-matter volumes were extracted from the same parcellation to provide complementary information on brain structure. Total intracranial volume (TIV) was estimated by FreeSurfer and used as a covariate in all volumetric analyses to account for individual differences in head size. All cortical thickness and volumetric outputs were subjected to the same quality control steps: automated surface/label reports, slice-by-slice visual inspection of tissue boundaries in native space, and verification of outliers flagged by FreeSurfer’s internal statistics. No manual edits were required for the reported analyses; scans failing basic QC would have been reprocessed or excluded. For statistical modeling, left and right hemisphere parcel measures were retained as separate regions of interest (ROIs) and exported to R/SPSS. ROI selection for hypothesis testing aligned with the a priori temporal, frontal, cingulate, and occipito-temporal regions implicated in aging and AD (e.g., lateral orbitofrontal cortex, temporal pole, posterior/caudal anterior cingulate, lingual gyrus; Dickerson et al., 2009; Bakkour et al., 2013). CT models adjusted for age, sex, education, and diagnosis; volumetric models additionally adjusted for TIV.

## Statistical analyses

### Primary models: time-window–specific hierarchical multiple regressions

To examine life-course effects while maintaining interpretability, we conducted time-window–specific hierarchical multiple regression models for each outcome across developmental periods TW1-TW6 (TW7 was not modeled; see below). For each outcome, covariates were entered in Block 1 (age, sex, education, and diagnostic group). For volumetric outcomes, TIV was included as an additional covariate in Block (1) The four dNA domain scores (COG, PHY, CRE, SOC) for the relevant TW were entered simultaneously in Block (2) This approach tested whether domain-specific engagement within each life period accounted for unique variance in later-life outcomes beyond demographic and clinical factors. Model assumptions, multicollinearity (VIF, condition indices), and influence (Cook’s distance) were checked for all reported models.

### Exploratory pattern characterization and focused summaries

A central goal was to identify which lifestyle domains, and which life periods (TW) may be most strongly associated with later-life outcomes. After modeling each TW separately, we examined the pattern of associations across all modeled windows (TW1-TW6) and domains. To present these observed patterns parsimoniously, we report focused summary models that highlight the single domain–TW predictor showing the strongest and most reliable association for each outcome. These focused models—re-estimated with only that significant predictor plus covariates—provide a descriptive summary of the most pronounced effects and are interpreted as exploratory, hypothesis-generating results rather than confirmatory tests.

### Mediation and moderation analyses

We examined whether cortical structure mediated the associations between dNA engagement and cognition (RAVLT and semantic fluency), and whether sex, education, or diagnostic factors moderated these relationships. Mediation analyses tested whether regional CT or volume statistically explained the link between early lifestyle engagement and later cognitive outcomes (for example CRE1 → left superior temporal CT → semantic fluency). Models were estimated with ordinary least squares regression and nonparametric bootstrap resampling (5,000 draws). We report the average causal mediation effect (ACME), the average direct effect (ADE), and the total effect with 95% confidence intervals. Mediation models adjusted for age, sex, education, diagnosis, and TIV when the mediator or outcome was volumetric. Moderation analyses tested whether sex, education, or diagnostic group (CN vs. MCI) modified the strength of observed associations. Interaction terms (Domain × Moderator; CT/Volume × Moderator) were added to the hierarchical regression models predicting both neuroimaging indices (CT and regional volumes) and neuropsychological outcomes (semantic fluency, RAVLT learning and delayed recall). Significant interactions were probed at ± 1 SD of education and stratified by sex and diagnostic group. All moderation models included age, sex, education, diagnosis, and TIV (for volumetric outcomes) as covariates.

### Statistical threshold, multiple comparisons correction, and interpretation

Statistical significance for all primary regression and mediation/moderation models was assessed using a False Discovery Rate (FDR) correction (Benjamini-Hochberg procedure) applied across all reported statistical tests within Tables 1 and 2. All tests were two-tailed, with the FDR-corrected alpha level (αFDR) set at 0.05. Given the modest sample size and the number of models tested, findings, particularly those outside the a priori hypotheses, should be interpreted with caution and are presented as hypothesis-generating. Analyses were conducted in IBM SPSS Statistics v30 and R 4.5.1 (RStudio).

**Table 1:** Demographics

Variable	CN (*n* = 39)	MCI (*n* = 19)	*p*-value
Age, M (SD)	72.3 (5.8)	74.1 (6.1)	0.312
Sex (% female)	56.4%	52.6%	0.776
Education, M (SD)	15.8 (2.6)	15.2 (2.9)	0.399
Amyloid-positive n (%)	5 (12.8%)	4 (20.0%)	0.468

**Table 2:** Significant HMR results — dNA → Cognitive

dNA	Dx	Cog Test	*N*	β	95% CI	*p*	FDR *p*	ΔR² Block	*p* (F-change)
COG1	All	Vegetables	58	0.410	[0.239,0.843]	0.001	0.003	0.154	0.001
COG1	CN	Vegetables	39	0.525	[0.239,0.811]	0.001	0.003	0.249	0.001
COG1	All	Animals	58	0.487	[0.203,0.771]	0.002	0.005	0.162	0.018
PHY1	All	RAVLT–Learn	55	0.417	[0.154,0.681]	0.003	0.006	0.233	0.007

Block 1 = age, sex, edu, diagnosis; Block 2 = dNA domains from the same time window (TW). dNA = dynamic Neurocognitive Adaptation; CN = cognitively normal; MCI = mild cognitive impairment; FDR = False Discovery Rate adjusted p-value (Benjamini-Hochberg procedure). Time windows: TW1 = 0–10 years, TW2 = 11–20 years, TW3 = 21–30 years, TW4 = 31–40 years, TW5 = 41–50 years, TW6 = 51–64 years

## Results

### Demographics

The CN and MCI groups were comparable on demographic characteristics and amyloid status, with no statistically significant differences in age, sex, years of education, or amyloid positivity (see Table 3 for detailed descriptive statistics and between-group comparisons). Differences between groups in self-reported life-course engagement (dNA scale scores) and neuropsychological test performance are reported in Supplementary Table 1. As shown, the two groups did not differ significantly in their reported engagement across dNA domains. In contrast, and consistent with diagnostic classification, the MCI group performed lower on MoCA and semantic fluency tests.

**Table 3:** Significant dNA → Neuro results (CT & Volumetric)

dNA	ROI	Dx	β	CI	*p*	FDR.*p*	ΔR.	*p* (F-change)
CRE1	L_CAC	All	0.314	[0.042,0.586]	0.028	0.036	0.079	0.028
PHY1	L_PCC	All	0.232	[0.021, 0.443]	0.036	0.043	0.052	0.036
CRE1	R_TP	All	0.335	[0.048, 0.621]	0.026	0.036	0.088	0.026
CRE3	R RMF	All	0.226	[0.008, 0.445]	0.048	0.053	0.041	0.048
COG4	R_LOFC_CT	All	0.469	[0.207, 0.730]	< 0.001	0.003	0.166	< 0.001
COG4	L Lingual	All	0.312	[0.057, 0.567]	0.020	0.033	0.073	0.020
COG4	R Lingual	All	0.413	[0.158, 0.668]	0.003	0.006	0.128	0.003
COG5	L Lingual	All	0.273	[0.016, 0.529]	0.042	0.046	0.057	0.042
COG5	R Lingual	All	0.477	[0.232, 0.721]	< 0.001	0.003	0.173	< 0.001

All neuroimaging results refer to regional brain volumes, with the sole exception of R_LOFC_CT, which denotes right lateral orbitofrontal cortex cortical thickness. Block 1 = age, sex, education, diagnosis, TIV; Block 2 = the four dNA domains from the same time window

### dNA - cognitive

**Fig. 1:**
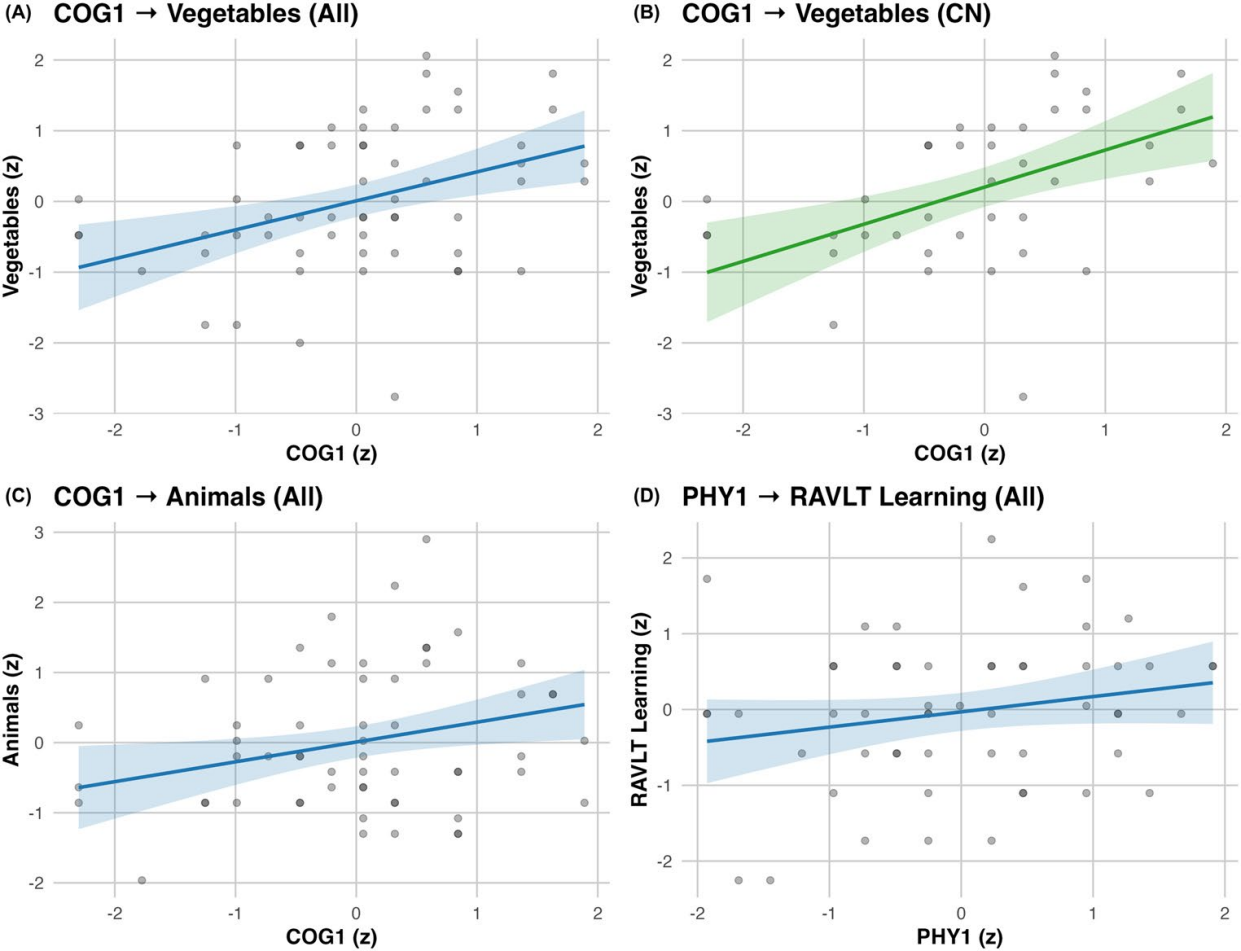
dNA COG1 and PHY1 associations with cognitive scores. Panels show partial regressions with adjusted fits (solid line) and 95% confidence intervals (shaded bands); axes are z-scores. Covariates: age, sex, education, and diagnosis. The CN-only panel excludes participants with MCI. Notes: Models are adjusted as above; no influential outliers; quadratic terms non-significant unless indicated in the text

Childhood (TW1, 0–10 y) was the only life period in which lifestyle engagement was correlated to cognition beyond covariates. In the TW1 model, COG1 was associated to semantic fluency for vegetables (β = 0.410, 95% CI [0.239, 0.843], *p* = .001; ΔR² = 0.154) and for animals (β = 0.487, 95% CI [0.203, 0.771], *p* = .002; ΔR² = 0.162). Within CN only, COG1 remained significant for vegetables (β = 0.525, 95% CI [0.239, 0.811], *p* = .001; ΔR² = 0.249). For episodic learning, PHY1 in TW1 was associated with RAVLT learning (β = 0.417, 95% CI [0.154, 0.681], *p* = .003; ΔR² = 0.233; Table 1; Fig. 1). No domain at any TW predicted RAVLT delayed recall. All models adjusted for age, sex, education, and diagnosis; values reflect the TW-specific block-2 change after adding the four domains.

All reported cognitive (neuropsychological) associations survived false discovery rate (FDR) correction (Table 1).

### dNA - neuro (imaging)

**Fig. 2:**
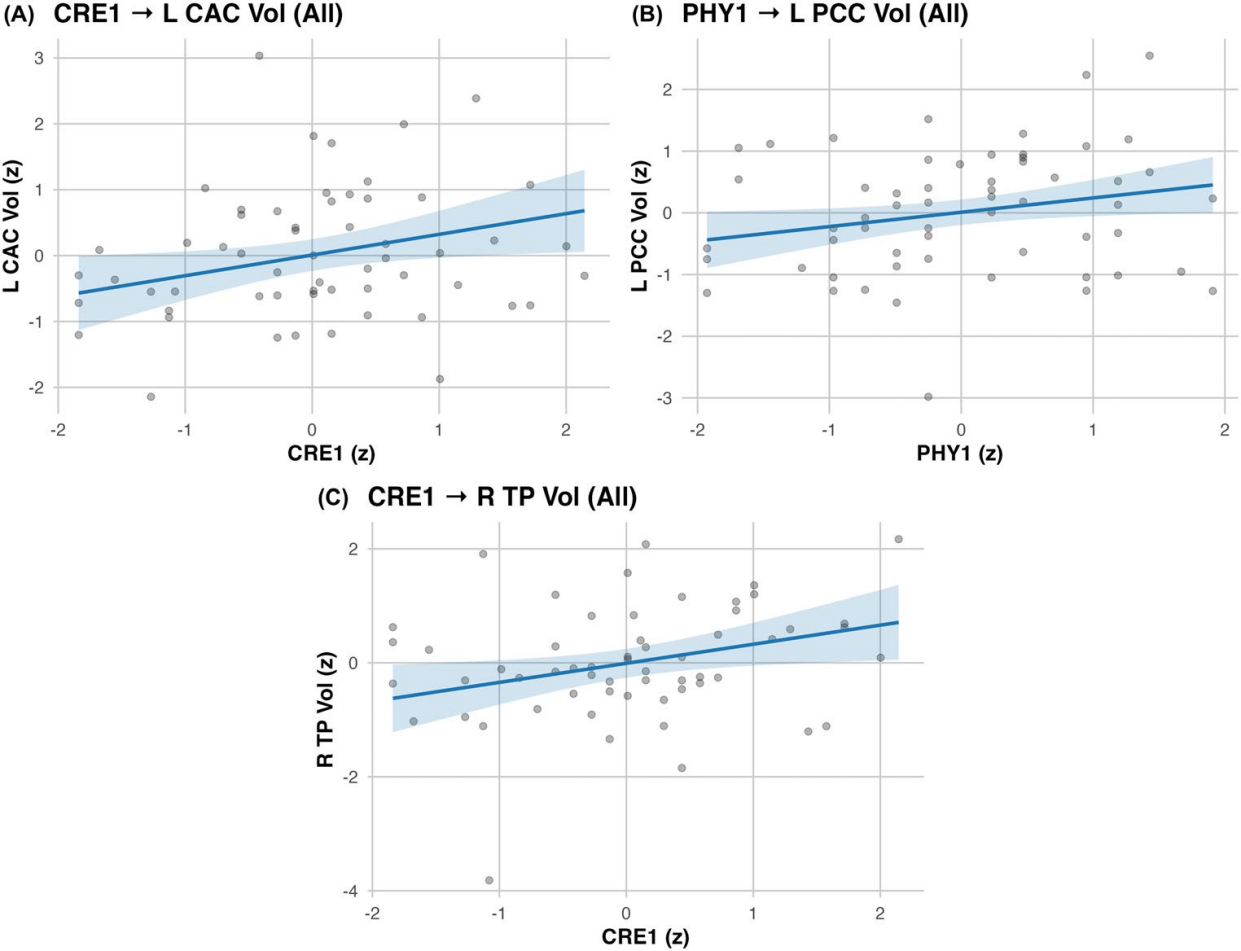
dNA PHY1 and CRE1 associations with brain structures. Panels show adjusted partial regressions of regional cortical volume on dNA scores with 95% confidence intervals. Axes are z-scores. Covariates: age, sex, education, and diagnosis; volume models additionally adjust for total intracranial volume (TIV)

**Fig. 3:**
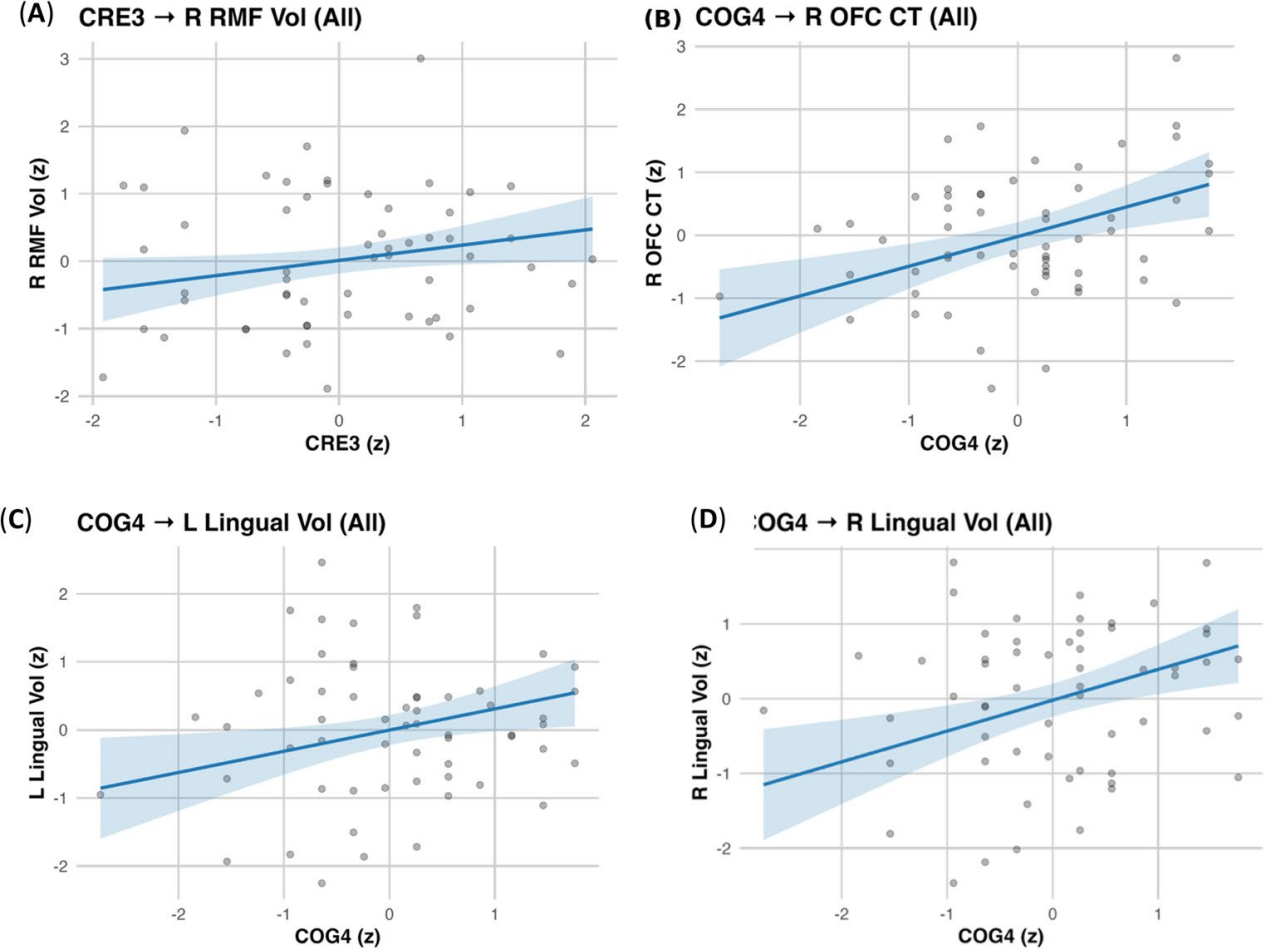
dNA CRE3 and COG4 associations with brain structures. Panels show adjusted partial regressions of regional cortical thickness or volume on dNA scores with 95% confidence intervals. Axes are z-scores. Covariates: age, sex, education, and diagnosis; volume models additionally adjust for total intracranial volume (TIV)

**Fig. 4:**
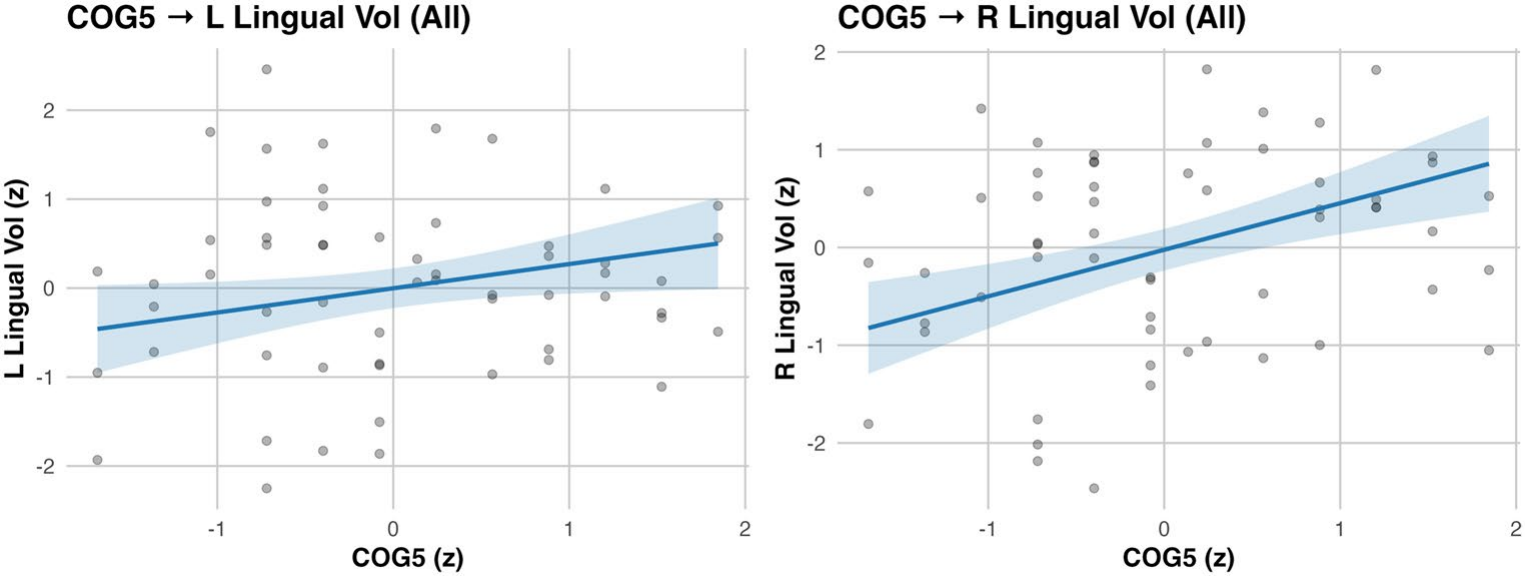
dNA COG5 associations with brain structures. Panels show adjusted partial regressions of regional cortical volume on dNA COG5 scores with 95% confidence intervals. Axes are z-scores. Covariates: age, sex, education, and diagnosis; volume models additionally adjust for total intracranial volume (TIV)

Childhood engagement showed significant volumetric associations. In TW1, CRE1 related to left caudal anterior cingulate volume (β = 0.314, 95% CI [0.042, 0.586], *p* = .028; ΔR² = 0.079), CRE1 to right temporal pole volume (β = 0.335, 95% CI [0.048, 0.621], *p* = .026; ΔR² = 0.088), and PHY1 to left posterior cingulate volume (β = 0.232, 95% CI [0.021, 0.443], *p* = .036; ΔR² = 0.052; Fig. 2). A TW3 and TW4 effect was also observed: CRE3 was associated with right rostral middle frontal volume (β = 0.226, 95% CI [0.008, 0.445], *p* = .048; ΔR² = 0.041). In TW4, COG4 was associated to right lateral orbitofrontal cortical thickness (β = 0.469, 95% CI [0.207, 0.730], *p* < .001; ΔR² = 0.166) and lingual volumes (left: β = 0.312, *p* = .020, ΔR² = 0.073; right: β = 0.413, *p* = .003, ΔR² = 0.128; Table 2; Fig. 3). In TW5, COG5 was linked to right lingual volume (β = 0.477, 95% CI [0.232, 0.721], *p* < .001; ΔR² = 0.173) and showed a smaller effect for left lingual volume (β = 0.273, *p* = .042; ΔR² = 0.057; Table 2; Fig. 4). All neuro models adjusted for age, sex, education, diagnosis, and for TIV in volumetric outcomes are summarized in Table 2. All reported neuroimaging associations survived FDR correction, except for a nominal CRE3–right rostral middle frontal association that did not remain significant after correction (Table 2).

### Mediation and moderation analyses

Sociodemographic mediation (education, sex, diagnosis): We tested education, sex, and diagnosis as mediators of significant dNA–neuroimaging and dNA–neuropsychological associations (covariates: age; plus TIV when volume was the outcome/mediator). Indirect effects were uniformly non-significant; all ACME 95% CIs included zero. For example, in the strongest volumetric association (right lingual volume × COG5, ΔR² = 0.173, *p* = .0004), education did not mediate the effect (ACME = − 0.005, 95% CI [− 0.075, 0.066], *p* = .89). Sex- and diagnosis-mediated paths were likewise non-significant across models (all ACME CIs spanning zero).

Brain mediation: To evaluate dNA → brain → cognition pathways, we ran bias-corrected bootstrapped mediation (5,000 resamples) using significant dNA–neuroimaging pairs as predictors and matched cognitive outcomes as dependents, controlling for age, sex, education, and TIV (for volumetric mediators). Across all models, indirect effects were non-significant (ACME CIs included zero). For instance, the indirect effect of COG5 on RAVLT delayed recall via right lingual volume was not significant (ACME = 0.006, 95% CI [− 0.018, 0.034], *p* = .41). Similar null mediation was observed for cortical thickness mediators (e.g., precuneus, lateral orbitofrontal, parahippocampal).

Moderation (sex, education, diagnosis): Interaction terms indicated no moderation of significant dNA–neuroimaging or dNA–cognitive associations by sex, education, or diagnosis: sex ps = 0.27–0.97; education ps = 0.28–0.99; diagnosis ps = 0.11–0.97 (all non-significant). Taken together, while direct associations between dNA domains and both neuroimaging and neuropsychological measures were significant and robust, these relationships were not mediated by education, sex, diagnosis, or regional brain structure, nor moderated by sex, education, or diagnosis.

## Discussion

In this study, we applied the dNA framework to link lifetime engagement with neurocognitive outcomes in aging, testing its associations with both cognitive performance and structural brain measures. Although our a priori hypothesis emphasized midlife as a sensitive period, the present findings highlight childhood as an additional, and in some domains stronger, window of association, underscoring the value of a life-course, exploratory framework. Childhood emerged as a particularly sensitive period, where early cognitive and physical activities were related to semantic fluency and episodic learning, as well as to volumetric measures in cingulate and temporal regions. Youth (TW3), adulthood (TW4), and middle age (TW5), also appear as key stages, partially confirming our recent results, regarding the importance of the middle age stage (Cieri et al. 2025b), with cognitive engagement associated with cortical thickness in frontal areas and volumes in occipito-temporal regions.

TW1was significantly involved in cognition. First, greater childhood cognitive engagement (COG1) was associated with better semantic fluency in later life. Second, childhood physical engagement (PHY1) was related to stronger episodic learning. These effects were observed after adjustment for age, sex, education, and diagnosis, and were not moderated by sex, education, or diagnosis. The association between COG1 and semantic fluency suggests the importance of early cognitive enrichment for later neurocognitive performance, as childhood experiences may scaffold semantic networks and establish a linguistic–executive foundation that supports fluency decades later. Experiences that challenge language, categorization, and executive control likely create durable representational maps and strategies for lexical access. Consistent with this view, recent evidence indicates that cognitively stimulating activities in childhood are associated with higher cognitive function in later life. Roberts et al. (2025) reported that enriching childhood activities predict better late-life cognition, supporting the idea that early engagement leaves a lasting imprint on cognitive trajectories. Similarly, Greenfield et al. (2021) and Ouyang et al. (2023) emphasized that access to learning opportunities and cognitive resources in early life shapes reserve and resilience decades later. The PHY1–learning association aligns with evidence that physical activity supports memory systems through neuroplastic mechanisms. Retrospective work linked regular early-life physical activity with better information processing speed in older age, even after adjusting for concurrent lifestyle factors (Dik et al., 2003).

TW1 was also associated to brain structure. Physical activity in childhood was related to larger volume in the left posterior cingulate cortex (PHY1 - PCC) and creative activities with right temporal pole (CRE1 - TP), regions deeply implicated in AD and in semantic/language networks. Converging functional and network analyses situate PCC and anterior temporal nodes (including TP) within interacting semantic and default-mode systems that support comprehension and concept integration across modalities (Binder et al., 2009; Jackson et al., 2019; Mesulam et al., 2013, 2021). Physical activity associated with PCC is consistent with PCC/default-mode sensitivity to lifestyle factors in aging. Observational and interventional studies link regular activity with greater gray matter volume and altered functional coupling within large-scale networks that include PCC (Gogniat et al., 2022; Pruzin et al., 2022). Large-sample MRI work further relates exercise to larger brain volumes in late life (Raji et al. 2024a, b).

Creative engagement in childhood (CRE1) was also associated with ACC volume, aligning with evidence implicating ACC in creative control (Beaty et al., 2016; Jung et al., 2013) and with training studies indicating experience-dependent plasticity in temporal association and connected fronto-parietal regions (Hervais-Adelman et al. 2017a, b, c; Hyde et al., 2009). Importantly, anterior cingulate cortex (ACC) hubs show altered functional connectivity in aging and MCI, underscoring the cingulate’s relevance to prodromal AD (Cera et al., 2019).

These findings support the view that early creative experience can shape temporal-lobe systems for language and multimodal integration, providing a mechanistic context for our TW1 associations in which childhood creative and physical engagement relate to temporal pole and cingulate morphometry—regions relevant to Alzheimer’s disease and central to semantic–memory networks.

Beyond childhood, TW3 to TW5 were linked to structural variation in prefrontal, orbitofrontal, and occipital regions. Our exploratory analysis found that CRE3 was associated with greater volume in the right rostral middle frontal gyrus. While this finding did not survive FDR correction for multiple comparisons, it is consistent with literature linking creative thinking to executive networks in prefrontal regions (Beaty et al., 2016). In parallel, cognitive engagement in midlife (COG4) was associated with the right lateral orbitofrontal CT, a region highly implicated early in AD pathology (Zhou et al., 2010). Cognitive activity across COG4–5 also predicted larger bilateral lingual gyrus volumes—a visual–semantic association region whose integrity has been tied to semantic fluency and visual memory in aging. Occipital thickening in midlife with higher physical fitness further supports this result (Tarumi et al., 2021). These findings align with life-course reserve models that place childhood and midlife as sensitive windows (Litkouhi et al., 2024; Livingston et al., 2024). Engagement in midlife cognitive, creative, and physical domains has been linked to healthier cortical thickness, gray matter integrity, and network connectivity (Ai et al., 2024; d’Arbeloff et al., 2021; Boa Sorte Silva et. al., 2024). Creative–temporal network hubs (e.g. temporal pole, cingulate) also covary structurally with creative experience (Hervais-Adelman et al. 2017a, b, c), offering mechanistic context for these frontal/occipital associations. Together, our result on TW3–TW5 suggest that the engagement later in life helps maintain structural integrity in executive and semantic–visual cortical systems vulnerable in aging and AD, partially consistent with ours (Cieri et al. 2025b) and others (Dohm-Hansen et al., 2024) recent findings, highlighting the importance of the middle age emphasizing the critical “middle-aging brain” period for adaptive and neurobiological change.

Together, these findings extend the dNA framework by showing that engagement beyond childhood—particularly during midlife—is associated with structural preservation in prefrontal–orbitofrontal and posterior visual cortices. These regions are central to networks supporting executive control, semantic integration, and visual memory, and their maintenance may represent one pathway through which lifestyle factors modulate neurocognitive aging trajectories.

Taken together, our results indicate that childhood has a fundamental role in shaping neurocognitive health trajectories. Early habits and activities may establish adaptive patterns that influence brain structure and function across the lifespan. Childhood appears pivotal for shaping neurocognitive trajectories, with early habits setting durable patterns in brain and cognition. Building on our prior dNA work (Cieri et al. 2025a, b a, b), an exposome-informed approach (Wild, 2005) aligns with the 2024 Lancet Commission (Livingston et al., 2024) indicating that up to ~ 45% of dementia may be preventable through modifiable factors from childhood through late life.

### Limitations

This study has several limitations. The sample was modest, reducing power and generalizability, including for CN–MCI contrasts. The cross-sectional design precludes causal inference; longitudinal follow-up is needed to trace how engagement across life periods shapes trajectories of brain structure and cognition. dNA scores relied on retrospective self-report and may be affected by recall bias—a limitation inherent to life-course assessments and only partially remediable; in practice, careful instrument design and triangulation with external records can mitigate (but not eliminate) this concern, and studies must ultimately rely on the credibility of the source reports Finally, we emphasized cortical thickness and volumetric; adding functional MRI, lifestyle factors (e.g., diet, socioeconomic context), and mental health measures would yield a more comprehensive characterization. Despite limitations, this study has clear strengths. Building on our prior dNA work—which specifies *what* forms of engagement (cognitive, physical, creative, social) and *when* across the lifespan—we extend the framework to neurobiological and cognitive endpoints by examining associations with regional volume and cortical thickness, and with episodic memory and semantic fluency; this integrative, life-course approach strengthens the bridge to the reserve–resilience field.

## Conclusions

This study applies the dNA framework to map *what* people engage in (cognitive, physical, creative, social domains) and *when* they do so across life-course time windows, relating those profiles to cognition, regional volumes, and cortical thickness. The approach identifies childhood (TW1) and adult-midlife (TW3–TW5) as sensitive periods: childhood physical and creative engagement align with cingulate and temporal morphometry and with later semantic and memory performance, while later cognitive engagement aligns with orbitofrontal thickness and lingual volumes. Together, these findings support the rationale for a life-course, exploratory approach such as dNA, which is specifically designed to capture dynamic, time-dependent associations rather than privileging a single developmental window. In practice, dNA provides a concise, time-resolved lens to link multidomain engagement to neurocognitive outcomes, specifying targets and windows for prevention.

## Supplementary Information

Below is the link to the electronic supplementary material.

Supplementary Material 1 (DOCX 18.1 KB)

## Data Availability

The datasets generated and/or analyzed during the current study are available from the corresponding author on reasonable request.

## Abbreviations

CAC: Caudal Anterior Cingulate
COG: Cognitive domain (of dNA)
CRE: Creative domain (of dNA)
CT: Cortical Thickness
dNA: Dynamic Neurocognitive Adaptation
IT: Inferior Temporal
LOF: Lateral Orbitofrontal
PHY: Physical domain (of dNA)
RLOFC: Right Lateral Orbitofrontal Cortex
RMF: Rostral Middle Frontal
SOC: Social domain (of dNA)
TIV: Total Intracranial Volume
TP: Temporal Pole
