# Supplementary material for: Dynamic neurocognitive adaptation: childhood and adult-midlife engagement associated with later-life brain structure and cognition in older adults with and without mild cognitive impairment
Source: Brain Imaging Behav. 2026 Mar 21;20(2):56. doi: 10.1007/s11682-026-01122-0 (PMC13004746; doi:10.1007/s11682-026-01122-0)
Supplement: Supplementary file 1 — Supplementary Material 1 (DOCX 18.1 KB) [file 11682_2026_1122_MOESM1_ESM.docx]

**Supplementary Table 1**

*Descriptive Statistics and Group Comparisons for dNA Domains and Neuropsychological Tests*

| **Measure** | **CN** M (SD) | **MCI** M (SD) | **t-test (df)** | **p** | **p (FDR)** |
| --- | --- | --- | --- | --- | --- |
| COG1 | 2.00 (0.97) | 2.12 (0.93) | -0.47 (57) | .642 | .690 |
| COG2 | 4.04 (1.35) | 3.52 (1.34) | 1.41 (57) | .163 | .525 |
| COG3 | 4.27 (1.31) | 3.68 (1.03) | 1.74 (57) | .086 | .313 |
| COG4 | 4.23 (1.39) | 3.89 (1.13) | 0.95 (57) | .348 | .631 |
| COG5 | 4.27 (1.27) | 3.92 (1.07) | 1.06 (57) | .293 | .608 |
| COG6 | 4.38 (1.50) | 3.89 (1.18) | 1.27 (57) | .210 | .587 |
| PHY1 | 1.95 (1.11) | 2.13 (0.90) | -0.62 (56) | .540 | .669 |
| PHY2 | 4.03 (1.66) | 3.62 (1.77) | 0.87 (57) | .386 | .638 |
| PHY3 | 4.26 (1.59) | 4.04 (1.80) | 0.49 (57) | .626 | .690 |
| PHY4 | 10.31 (3.85) | 10.60 (3.57) | -0.28 (57) | .778 | .778 |
| PHY5 | 3.88 (1.62) | 4.18 (1.31) | -0.71 (57) | .480 | .638 |
| PHY6 | 3.53 (1.72) | 3.80 (1.43) | -0.60 (57) | .553 | .669 |
| CRE1 | 1.45 (0.87) | 1.94 (0.82) | -2.09 (57) | .041 | .285 |
| CRE2 | 3.64 (1.33) | 3.92 (1.45) | -0.75 (57) | .458 | .638 |
| CRE3 | 3.57 (1.39) | 3.84 (1.23) | -0.71 (57) | .480 | .638 |
| CRE4 | 3.52 (1.34) | 3.86 (1.12) | -0.99 (57) | .324 | .627 |
| CRE5 | 3.50 (1.29) | 3.92 (1.16) | -1.23 (57) | .222 | .587 |
| CRE6 | 3.43 (1.40) | 3.72 (0.88) | -0.84 (57) | .403 | .638 |
| SOC1 | 3.01 (0.95) | 3.28 (0.73) | -1.13 (57) | .263 | .588 |
| SOC2 | 5.36 (1.20) | 5.58 (1.08) | -0.70 (57) | .484 | .638 |
| SOC3 | 9.85 (2.43) | 10.18 (2.07) | -0.51 (57) | .614 | .690 |
| SOC4 | 4.98 (0.92) | 5.40 (0.68) | -1.79 (57) | .079 | .313 |
| SOC5 | 5.02 (0.86) | 5.45 (0.69) | -1.93 (57) | .059 | .285 |
| SOC6 | 4.91 (0.97) | 5.20 (0.88) | -1.14 (57) | .261 | .588 |
| MoCA | 21.56 (5.92) | 18.05 (4.08) | 2.38 (57) | .021 | .201 |
| RAVLT Learning | 51.27 (10.04) | 52.05 (8.77) | -0.29 (54) | .775 | .778 |
| SemFlu-Anim | 18.57 (3.89) | 14.74 (4.15) | 3.01 (38) | .005 | .010 |
| SemFlu-Veget | 14.32 (2.87) | 11.52 (3.89) | 2.59 (38) | .014 | .014 |

Note. dNA = dynamic Neurocognitive Adaptation; CN = cognitively normal; MCI = mild cognitive impairment; FDR = False Discovery Rate adjusted p-value (Benjamini-Hochberg procedure). Time windows: TW1 = 0-10 years, TW2 = 11-20 years, TW3 = 21-30 years, TW4 = 31-40 years, TW5 = 41-50 years, TW6 = 51-64 years.
